# A Decentralized Fuzzy C-Means-Based Energy-Efficient Routing Protocol for Wireless Sensor Networks

**DOI:** 10.1155/2014/647281

**Published:** 2014-08-05

**Authors:** Osama Moh'd Alia

**Affiliations:** Faculty of Computers and Information Technology, University of Tabuk, P.O. Box 741, Tabuk 71491, Saudi Arabia

## Abstract

Energy conservation in wireless sensor networks (WSNs) is a vital consideration when designing wireless networking protocols. In this paper, we propose a Decentralized Fuzzy Clustering Protocol, named DCFP, which minimizes total network energy dissipation to promote maximum network lifetime. The process of constructing the infrastructure for a given WSN is performed only once at the beginning of the protocol at a base station, which remains unchanged throughout the network's lifetime. In this initial construction step, a fuzzy C-means algorithm is adopted to allocate sensor nodes into their most appropriate clusters. Subsequently, the protocol runs its rounds where each round is divided into a CH-Election phase and a Data Transmission phase. In the CH-Election phase, the election of new cluster heads is done locally in each cluster where a new multicriteria objective function is proposed to enhance the quality of elected cluster heads. In the Data Transmission phase, the sensing and data transmission from each sensor node to their respective cluster head is performed and cluster heads in turn aggregate and send the sensed data to the base station. Simulation results demonstrate that the proposed protocol improves network lifetime, data delivery, and energy consumption compared to other well-known energy-efficient protocols.

## 1. Introduction

Wireless sensor networks (WSNs) are a collection of sensors used to observe physical and/or environmental phenomenon such as heat, humidity, vibration, light, and pressure. A WSN consists of sensor nodes, which are equipped with sensing capabilities, wireless communication interfaces, and limited processing and energy resources. One or more powerful base stations (BS) serve as the final destination of the sensed data [1]. The potential applications of WSNs in civilian and military domains include environmental monitoring, surveillance, healthcare, traffic control, object tracking, and so forth [2]. For instance, a WSN can be used in agriculture to monitor water levels, temperature, and humidity for a particular plantation site.

Passing sensory data to the BS requires energy. Therefore, in order to ensure the WNS's operational longevity, energy consumption is a critical consideration when designing WSN routing protocols. Furthermore, since sensor nodes are often in difficult-to-reach locations, constant replacement of batteries (the power source for the nodes) is impractical.

Recently, instead of directly tackling the physical infrastructure of WSNs, researchers have opted to solve the abovementioned issues using computer algorithms [3–5]. Cluster-based techniques are one of the most innovative techniques in this aspect, where they have been proven to be scalable and efficient [5]. In the context of WSNs, clustering is able to assemble sensor nodes into clusters (groups), where each cluster has a cluster head (CH). The CH plays a vital role in the reception of sensed data from sensors related to its cluster, where this data is then filtered to remove redundancy before being relayed to the BS. Through this infrastructure, efficient utilization of the energy resources is possible because all the data being transmitted to the BS is significantly reduced. Moreover, the transmission distance from cluster member nodes with their CHs is less than the transmission distance from these nodes to the BS, which also reduces energy consumption. In addition to all of that, the process of rotating the role of being a CH between cluster member nodes further reduces energy consumption since non-CH member nodes can go into sleep mode for a longer period of time.

In recent years, many hierarchical clustering routing protocols have been developed for WSNs. Among the common ones are Low-energy Adaptive Clustering Hierarchy (LEACH) [6], Hybrid Energy-Efficient Distributed clustering (HEED) [7], Hierarchical Cluster-based Routing (HCR) [8], Power-Efficient Gathering in Sensor Information Systems (PEGASIS) [9], Threshold sensitive Energy Efficient sensor Network (TEEN) [10], and Stable Election Protocol (SEP) [11]. For other protocols, readers can be directed to the work by [5]. Among these protocols, fuzzy C-means-based routing protocols are considered good solutions to improve the network lifetime and optimize the cluster structure [12]. The fuzzy C-means algorithm (FCM) was proposed by Bezdek [13] and has been used in cluster analysis, pattern recognition, image processing, and so forth. In the context of WSNs, this algorithm assigns each sensor node to a cluster with a degree of membership. In the literature, protocols such as [12, 14–16] were proposed to overcome the issue of uneven distribution of sensor nodes related with the application of protocols like LEACH. A uniform creation of clusters in randomly deployed sensor networks was performed where the total spatial distance among the sensor nodes within each cluster was minimized [12]. However, these protocols are centralized hierarchical protocols where the cluster formation and CH election are carried out at the BS. This in turn adversely effects the network's energy consumption since the residual energy and the geographical location of all alive sensor nodes are delivered to the BS at the end of each round. Furthermore, the CH election mechanism is only based on the highest residual energy factor for the non-CH competitors within each cluster, as mentioned in [12]. This can lead to the election of an inappropriate CH where its distance is not optimal from the rest of the sensors in the same cluster and to its BS.

In this paper, we propose a Decentralized Fuzzy Clustering Protocol (DFCP) for energy-efficient WSNs. DFCP is meant to minimize total network energy dissipation, while extending the network's lifetime. The process of constructing an infrastructure for a given WSN is performed only once at the beginning of the protocol at a BS, which remains unchanged throughout the network's lifetime. In this initial construction step, the fuzzy C-means algorithm allocates sensor nodes into their most appropriate clusters. Subsequently, the protocol runs its rounds (iterates) where each round is divided into a CH-Election phase and a Data Transmission phase. In the CH-Election phase, the election of new cluster heads is done locally in each cluster where a new multicriteria objective function is proposed to enhance the quality of the elected cluster heads. In the Data Transmission phase, sensing and data transmission from each sensor node to their cluster head is performed where the cluster heads in turn aggregate and send the sensed data to the BS.

The rest of the paper is organized as follows.  Section 2 provides the preliminaries of the network along with its radio model. A detailed description of the proposed protocol using the fuzzy C-means algorithm is given in Section 3. The simulation study of the proposed protocol is presented in Section 4. We conclude our findings in Section 5.

## 2. Preliminaries

This section presents the assumptions and radio energy model of the network under consideration.

### 2.1. Assumption

- The base station is located far from the sensor nodes and is immobile.
- All nodes in the network are homogeneous and energy constrained.
- Symmetric propagation channel is employed.
- Nodes have location information with respective energy levels.
- Nodes are immobile.

### 2.2. Radio Energy Model

With regards to the radio energy model used in this work, which is similar to the model proposed in [6], the transmitter dissipates energy to run the radio electronics and the power amplifier, and the receiver dissipates energy to run the radio electronics. The energy consumption for transmitting a *b*-bit message over a distance *d* is

(1) ETx=Eelec×b+Efs×b×d2, d<d0,

(2) ETx=Eelec×b+Emp×b×d4, d≥d0,

and for receiving this message, the energy consumption is

(3) $$\begin{array}{c} E_{\text{Rx}}=E_{\text{elec}}\times b, \end{array}$$

where *E*_elec_ is the energy to operate the transceiver circuit; *E*_fs_ and *E*_mp_ are the energy expenditures for transmitting one-bit of data to achieve an acceptable bit error rate depending on the transmission distance in the case of the free space model and multipath fading model [6]. If the transmission distance is less than a threshold *d*_0_, the free space model is applied as in (1); otherwise, the multipath model is used as in (2). The threshold *d*_0_ is calculated as

(4) $$\begin{array}{c} d_{0}=\sqrt{\left(\frac{E_{\text{fs}}}{E_{\text{mp}}}\right)}. \end{array}$$

Data aggregation, which is performed by the CH to reduce the total amount of sent data is calculated as *E*_da_ = 5 nJ/bit/message. This is based on the assumption that the overall data collected by a cluster of *n*-nodes, where each node collects *b*-bits of data, can be compressed to *b*-bits regardless of the number of nodes in that cluster. Another parameter is also taken into consideration, which is related to the energy consumption of CH when a new CH is elected for the next round. We propose a new energy consumption parameter *E*_CH_Elec_, which is responsible to calculate the CH election energy expenditure of CH and is set to *E*_CH_Elec_ = 5 nJ × No.Above.Ave, where No.Above.Ave represents the number of candidate CHs within a cluster that are above the average energy of alive nodes.

## 3. The Proposed Protocol

**Figure 1:**
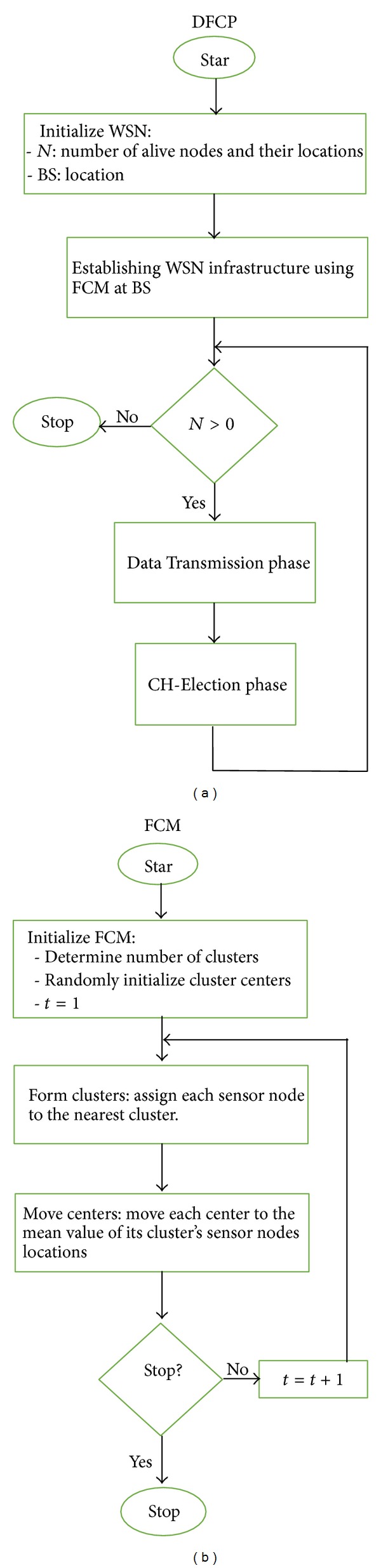
(a) An overview of the proposed protocol DFCP. (b) FCM algorithm.

In this section, we present the proposed fuzzy clustering protocol for the energy conservation problem in WSNs. The protocol is a decentralized fuzzy clustering protocol where in the base station the infrastructure of a given WSN is established by the FCM algorithm. In this initial construction step, FCM is responsible of allocating sensor nodes into their most appropriate clusters based on their geographical locations. This process is performed only once at the beginning of the protocol at the base station, which remains unchanged throughout the network's lifetime. In other words, the infrastructure of the network is permanent once it is designed where no sensor node can be moved from one cluster to another. Subsequently, the protocol is iterative and each round is divided into a CH-Election phase and a Data Transmission phase. In the CH-Election phase, the election of the new cluster heads is done locally in each cluster where a new multicriteria objective function is proposed to enhance the quality of the elected cluster heads. In the Data Transmission phase, sensing and data transmission from each sensor node to their cluster head is performed, where the cluster heads in turn aggregate and send the sensed data to the base station.  Figure 1(a) provides an overview of the proposed protocol, where its detailed description is given in the following section.

### 3.1. Establishing WSN Infrastructure Using FCM

Clustering is an unsupervised learning technique for grouping similar data points according to some measure of similarity that maximizes the intercluster similarity while minimizing the intracluster similarity [13]. A clustering algorithm of the fuzzy partitioning type is performed on a set of *N* data points *X* = {*x*_1_, *x*_2_,…, *x*_*N*_}, where each *x*_*i*_ ∈ *R* ^*f*^ is a feature vector consisting of *f* real-valued measurements describing the features of the data point *x*_*i*_. Fuzzy clusters, *c*, of the data points can be represented by a fuzzy membership matrix called a fuzzy partition *U* = [*u*_*ij*_]_*c*×*n*_ where *u*_*ij*_ represents the fuzzy membership of the *i*th data point to the *j*th fuzzy cluster. Every data point therefore belongs to a particular (possibly null) fuzzy cluster based on the calculated degree of membership.

Given a WSN that consists of *N*-sensor nodes randomly distributed over an area of *M* × *M* meters, these nodes send a short message (termed an advertisement message) to the BS containing information of their respective geographical locations. Based on the information received from the sensor nodes, the BS computes the cluster centers and allocates sensor nodes *X* to the clusters *c* by applying the FCM algorithm. Each node is assigned a degree of membership *u*_*ij*_ to a cluster *C*_*j*_ rather than completely being a member of just one cluster.

FCM is an iterative procedure that aims to locally minimize the following objective function:

(5) $$\begin{array}{c} J_{m}=\overset{c}{\underset{j=1}{\sum }}\overset{n}{\underset{i=1}{\sum }}u_{ij}^{m}{||x_{i}-v_{j}||}^{2}, \end{array}$$

where {*v*_*j*_}_*j*=1_^*c*^ are the centers of the clusters *c* and the array *u*_*ij*_ represents the fuzzy membership matrix, *U* ∈ *M*_*fcn*_ as in (6), ||·|| denotes an inner-product norm (e.g., Euclidean distance) from the sensor node *x*_*i*_ to the *j*th cluster center, and the parameter *m* ∈ [1, *∞*) is a weighting exponent on each fuzzy membership that determines the amount of fuzziness of the resulting classification:

(6) Mfcn={U∈Rc×n ∣ ∑j=1cuij=1, 0<∑i=1nuij<n,uij∈[0,1];1≤j≤c;1≤i≤n}.

FCM's steps shown in Figure 1(b) can be summarized as follows.

(1) Select the number of fuzzy clusters *c*.
(2) Select the initial cluster centers *v*_1_, *v*_2_,…, *v*_*c*_.
(3) Compute the elements of the fuzzy partition matrix using
  (7) $$\begin{array}{c} u_{ij}=\frac{1}{\sum_{k=1}^{c}{\left(||x_{i}-v_{j}||/||x_{i}-v_{k}||\right)}^{2/\left(m-1\right)}}. \end{array}$$
(4) Compute the cluster centers using
  (8) $$\begin{array}{c} v_{j}=\frac{\sum_{i=1}^{n}u_{ij}^{m}\cdot x_{i}}{\sum_{i=1}^{n}u_{ij}^{m}}. \end{array}$$
(5) Repeat Steps (3) and (4) until the number of iterations *t* exceeds a given limit, or until a termination criterion is satisfied:
  (9) $$\begin{array}{c} ||V_{\text{new}}-V_{\text{old}}||<E, \end{array}$$
  where *E* = 0.001.

After FCM forms the clusters, the closest sensor node to a particular cluster center is chosen to be a CH, as the location of the cluster center within a cluster is the most appropriate location to be a CH. This is because the cluster center mediates all sensor nodes within the cluster and this in turn reduces the amount of energy required by cluster nodes to send data. Furthermore, and in this stage, all nodes have almost the same energy level, which is consistent with the assumption made in Section 2.1. Hence, no node has higher priority to become a CH except if it is within close proximity to the cluster center. At this point, a* join* message is being sent to every sensor node in the network containing the information of the cluster it belongs to as well as the time schedule to transfer the data. Once the* join* message reaches a sensor node, the node extracts the network information from this message (such as the CH identification and transmission time schedule) and stores this information in its memory for forwarding during the Data Transmission phase.

After the WSN infrastructure is established, the protocol runs its rounds and each round is divided into the aforementioned CH-Election and Data Transmission phases. The following provides a description of these two phases.

*Phase 1: CH-Election Phase.* After the infrastructure of the WSN is developed by FCM in the base station, the CHs election process for the upcoming rounds is done locally in each cluster. The current CHs (the CHs that are elected from the previous round) will calculate the average energy level of all alive nodes in its cluster. Information about sensed data along with location and residual energy of each node will be the message content sent by nodes to their respective CH. With knowledge of the energy information, only nodes that have residual energy higher than the average level qualify as a CH candidate, cd_*i*_ ∈ CD_*c*_. The competition between candidate nodes to be a CH is based on the following factors:

- the residual energy in the candidate node;
- the location of each candidate node within a cluster; and
- the location of each candidate node with regards to the BS.

These factors are the main components of our proposed objective function that is used in the election process of CHs. The proposed objective function is described as follows (The early version of the proposed objective function was presented in [17]):

(10) $$\begin{array}{c} {\text{CH}}_{\text{obj}}=\underset{\forall {\text{cd}}_{i}\in {\text{CD}}_{c}}{\max}\left\{\frac{E_{{\text{cd}}_{i}}\times q}{\alpha \times f_{1}+\left(1-\alpha \right)\times f_{2}}\right\}, \end{array}$$

where

(11) f1=∑j=1nalive||nodejc−cdi||,f2=||cdi−BS||.

In this objective function, *E*_cd_*i*__ is the residual energy of the candidate cluster head cd_*i*_∈ cluster *C*_*j*_. *q*, which is set as *q* = 1000, is a constant term for a particular WSN and is used to avoid the objective function value from approaching zero. *f*_1_ is the Euclidean distance of all alive nodes in a particular cluster *C*_*j*_ to their candidate cluster head cd_*i*_. *f*_2_ is the Euclidean distance of the candidate cluster head cd_*i*_ to the BS. The constant *α* is the influence of *f*_1_ and *f*_2_ on the objective function. This objective function tends to minimize the intracluster distance (compactness) between sensor nodes and their CH, which in turn minimizes the energy required to pass the sensed data from each node to their CH. Furthermore, the objective function also tends to minimize the distance between CH and the BS, which in turn minimizes the energy required to pass the aggregated data from each CH to their BS. Therefore, finding the maximum value of the objective function CH_obj_ in each round of the proposed protocol for each cluster *C*_*j*_ is desired and indicates that the candidate cluster head cd_*i*_ is the best among other candidate competitors.

After the optimal CHs are selected, a* join* message is sent by the current CHs to all alive sensor nodes in their respective clusters, which contains the information of the new CHs as well as the time schedule to transfer the data. Once the* join* message reaches a sensor node, the node extracts the new CH identifier and transmission time schedule and stores this information in its memory to forward data during the Data Transmission phase.

*Phase 2: Data Transmission Phase.* Once all nodes receive the* join* message, and the transmission schedule is initialized, the sensor nodes activate their radio component for a very short period of time to perform data sensing and transmission to the CHs. At that time, the CHs must be awake to receive the data from the nodes in their clusters. Once the CHs receive all the data, they perform data aggregation where all individual signals in each cluster are combined into a single representative signal. This process, as assumed in this study, is to enhance the common signal and reduce the uncorrelated noise among the signals. The resultant data are sent from the CHs to the BS. This reduces the amount of information being transferred, hence also reducing energy consumption.

Both the CH-Election and Data Transmission phases are repeated in each round of the proposed protocol throughout the network's lifetime.

### 3.2. Complexity Analysis

The time complexity analysis of the proposed protocol is presented in this section. As mentioned earlier, the proposed DCFP protocol consists of two parts. In the first part, FCM constructs the infrastructure for the given WSN and is run just once at the beginning of the protocol. As reported by Hore et al. [18], the time complexity of the FCM is *O*(*ndc* ^2^ *i*), where *n* is the number of sensor nodes, *d* is the number of dimensions (set to 2 in our study, which represents the *x*- and *y*-axis location of each sensor node), *c* is the number of clusters, and *i* is the number of iterations of FCM over all nodes of the given WSN. Meanwhile, the amount of time to execute one complete round of the second part of DCFP is the time to complete the Data Transmission and CH-Election phases. By carefully examining these two phases, it can be seen that the CH-Election phase dominates the overall time. Thus, the analysis is focused on this phase and the equation given in (10). By a closer look into this equation and equations related to (11), we can find that the time complexity of this phase is *O*(*ndc* ^2^) as in FCM algorithm except the variable *i* that represents the number of iterations of FCM. It is worth mentioning here that *n* represents the number of alive sensor nodes in the given WSN. From the analysis above, the time complexity of the proposed DCFP is *O*(*n*) which is all far lower than LEACH [6] (at least *O*(*n* ^2^) as reported by [19]). The C-FCM protocol [12] has the same time complexity of DCFP, except that the process of cluster formation by FCM is repeated periodically during the network operation, which in turn adversely affects the performance and effectiveness of the protocol.

## 4. Simulation Results

### 4.1. Experimental Setup

**Figure 2:**
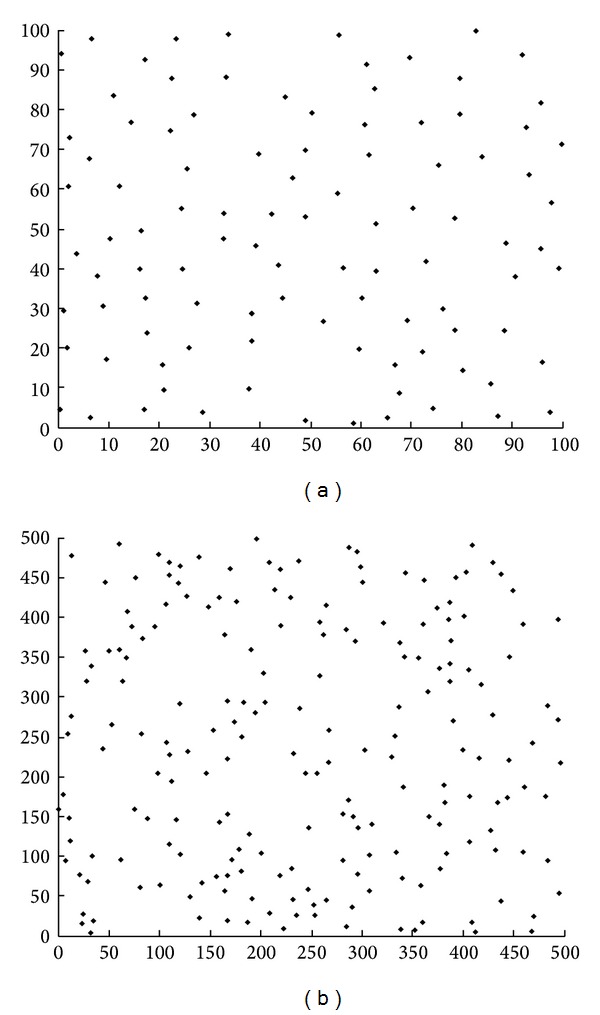
(a) Unclustered 100 sensor nodes in 100 m × 100 m WSN. (b) Unclustered 200 sensor nodes in 500 m × 500 m WSN.

In order to evaluate the proposed DFCP protocol, two different simulations were run using MATLAB version R2010a. The first simulation was done with 100-sensor nodes scattered randomly across a 100 m × 100 m network as shown in Figure 2(a), while the second simulation was done with 200 sensor nodes scattered randomly across a 500 m × 500 m network as shown in Figure 2(b). These simulations were performed to measure the performance of the DFCP compared to other existing protocols, when different settings are used for a simulated network.

In these two simulations, no two nodes can be in the same location. This means that the horizontal and vertical coordinates of each sensor are randomly selected between 0 and the maximum value of the dimension (i.e., 100 for the first simulation and 500 for the second simulation). The allowed minimum distance between each sensor node is set to be 6 meters in the first simulation and 10 meters in the second simulation. The BS location for the first simulation is set to be in (50,175) while in the second simulation is set to be in (500,575).

The coefficient *α* in (10) is set to *α* = 0.75 in both simulations to give the compactness factor more influence than the location of the candidate cluster head cd_*i*_ with regards to the BS. The radio energy parameters used in both simulations are set as *E*_elec_ = 50 pJ/bit, *E*_fs_ = 10 pJ/bit/m^2^, and *E*_mp_ = 0.0013 pJ/bit/m^4^ [6]. Each data message is set to *b* = 500 bytes/message, and the packet header for each type of packet is 25 bytes long.  Table 1 summarizes the network setting for both simulations.

Since the FCM algorithm requires the number of clusters to be determined* a priori*, we initialized the initial number of clusters to 5. The maximum iteration for the FCM algorithm is set to *t* = 100. The parameter *m* that determines the amount of fuzziness of the resulting cluster assignment is set to 2.

DFCP's capability and efficiency are evaluated by comparing it with another FCM-based energy efficient protocol proposed in [12]. The focus of this work is to measure the benefits of using the decentralized technique on top of the existing algorithm. Furthermore, the very well-known LEACH protocol is also presented in this study, where it is the second protocol that is compared with DFCP. The two other simulated protocols used as comparison with this work are described as follows.

- C-FCM [12]: this is a centralized clustering protocol using FCM. The C-FCM protocol consists of two phases. The first phase is the setup phase, which performs two tasks: (a) cluster formation and (b) CH selection. The second phase is the Data Transmission phase, which performs data gathering, aggregation, and sending from sensor nodes to their CHs and then to the BS. In each round of C-FCM, cluster formation is performed at the BS where the centralized FCM algorithm allocates sensor nodes to their appropriate cluster based on their geographical locations. The CH selection process on the other hand is based on the highest residual energy of the eligible nodes within each cluster.
- LEACH [6]: this is a distributed hierarchical clustering protocol that forms clusters of sensor nodes based on the received signal strength. Each CH acts as routers to the BS. Similar to DFCP, data aggregation and transmission is done by the CHs. The CH's role is randomly changed between nodes, where each node chooses a random number between 0 and 1. The node becomes a CH if the selected random number is less than a specified threshold *ρ*.

### 4.2. Results and Analysis

**Figure 3:**
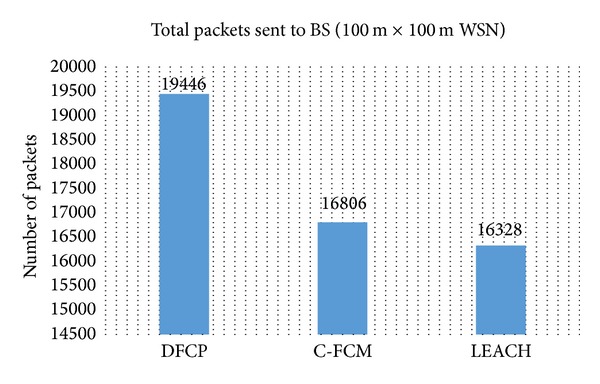
Total data delivered to the BS in 100 m × 100 m WSN.

**Figure 4:**
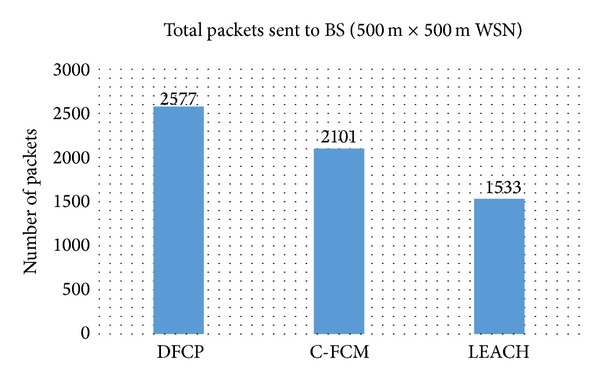
Total data delivered to the BS in 500 m × 500 m WSN.

The performance of the DFCP protocol in terms of its capability to deliver data to the BS and energy efficiency is compared with C-FCM and LEACH. Figures 3 and 4 show the total data received by the BS for the different simulated networks. They show that for both cases of different network area, DFCP can achieve higher data delivery compared to C-FCM and LEACH. The improvement achieved over C-FCM is about 16% for the first simulation. Meanwhile, the benefit of using DFCP is more significant when bigger network area is used, where the improvement is approximately 23% better compared to C-FCM. These results are also true when comparing with LEACH. The improvement in data delivery over LEACH is about 19% for the first simulation. Meanwhile, the benefit of DFCP is more significant in the second simulation with a larger network area, where the improvement in data delivery is approximately 68% better compared to LEACH. It is also observed that when the simulated network gets bigger, the energy required for communication increases as well. This is because, as the network area gets bigger, the density of the network decreases. Consequently, the distance between sensor nodes and the CHs, as well as the distance between CHs and the BS, becomes further. Therefore, more energy is expended causing less data being delivered to the BS. For instance, Figures 3 and 4 show the number of packets delivered to the BS by DFCP, with 19,446 packets for the 100 m × 100 m area network simulation, compared to 2,577 packets for the 500 m × 500 m area network simulation. The difference is obvious especially when the initial energy for each node in both simulations is known to be the same (e.g., 2 J). However, it can be seen that in both the 100 m × 100 m and 500 m × 500 m cases DFCP exploits the network energy at almost rate compared to the other protocols, which in turn results in higher data delivery. Thus, DFCP is a worthy approach to utilize network energy resources efficiently.

**Figure 5:**
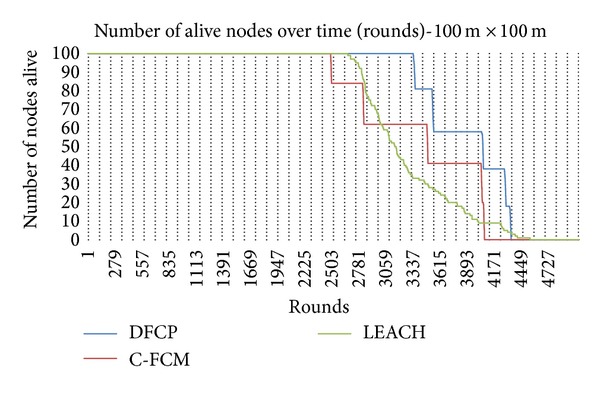
Network lifetime for 100 m × 100 m WSN.

**Figure 6:**
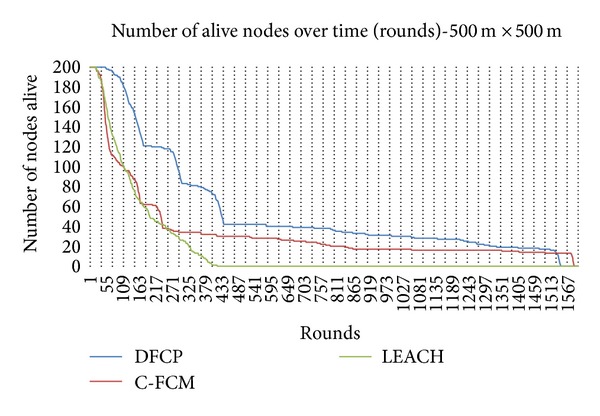
Network lifetime for 500 m × 500 m WSN.

To further illustrate the efficiency of DFCP, a demonstration of the system lifetime, defined by the number of nodes remaining alive throughout the entire duration of the simulation, is presented.  Figure 5 shows the network lifetime according to the percentage of sensor nodes dying for the network area 100 m × 100 m, while Figure 6 shows the similar results for the 500 m × 500 m case. These figures show the performance of the C-FCM and LEACH protocols compared with DFCP in terms of the number of rounds before the occurrence of a first dead node. It can be seen that the network lifetime for our proposed protocol is significantly better compared to C-FCM and LEACH. It is also observed the adverse effects of the bigger area network on the performance of the competitors' protocols. The bigger the network area, the bigger the energy required for communication; thus, the number of alive nodes throughout the entire duration of the simulation is adversely affected.

**Figure 7:**
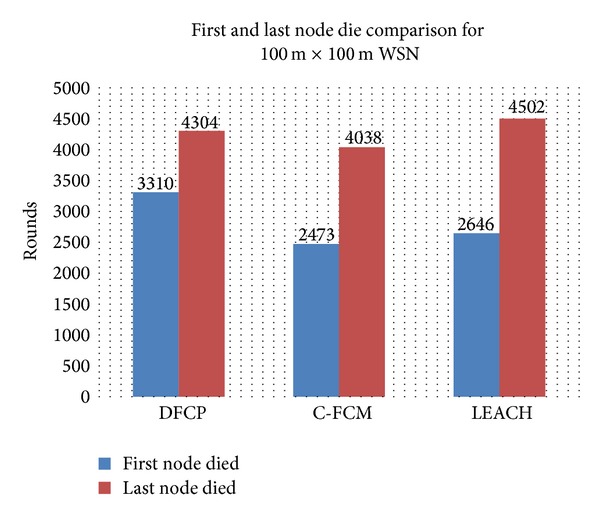
The first and last node die comparison for 100 m × 100 m area network.

**Figure 8:**
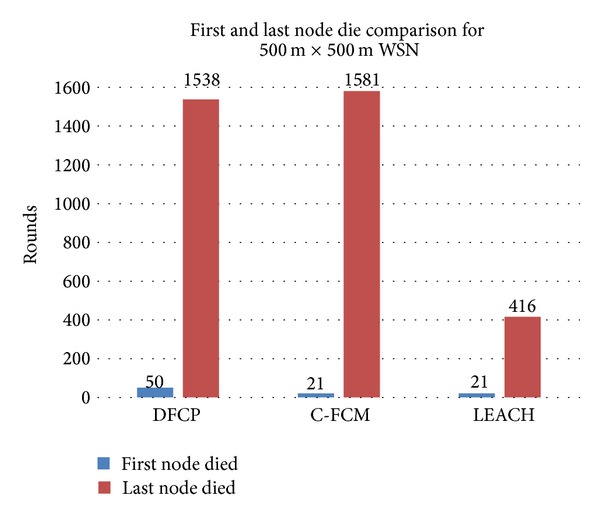
The first and last node die comparison for 500 m × 500 m area network.

Figures 7 and 8 show how the C-FCM and LEACH protocols compare with DFCP in terms of the number of rounds before the occurrence of a first dead node, as well as the number of rounds until the last dead node. It can be seen from both figures in both simulations that the network lifetime for DFCP is significantly better compared to C-FCM and LEACH.  Figure 7, which represents the simulation in the 100 m × 100 m area network, shows that the first node died after 2473 rounds for C-FCM and after 2646 rounds for LEACH, while the first node died after 3310 rounds for DFCP, which is approximately a 34% and 25% improvement of network lifetime, respectively.  Figure 7 also shows that the last node died after 4038 rounds for C-FCM and after 4502 rounds for LEACH, while for DFCP, it occurred after 4304 rounds. This is approximately a 7% improvement of network lifetime compared to C-FCM. The LEACH protocol performed better than DFCP performance in terms of the last dead node. However, this did not cause any improvement of the data delivered to the BS factor as represented in Figure 3. This result also confirms what has been mentioned in the introduction part of this paper that LEACH may suffer from the uneven distribution of sensor nodes in the simulated network. For the simulation of the 500 m × 500 m area network, Figure 8 shows that both DFCP and C-FCM have better performances in terms of the last dead node over LEACH, where both protocols approximately improved network performance by 280% compared to LEACH. Figure 8 also shows that all competitors' protocols have almost the same level of network performance in terms of the first dead node.

## 5. Conclusion

In WSNs, it is very important to develop routing protocols that can conserve energy of the nodes as much as possible to improve network lifetime. This led us to design a decentralized FCM-based protocol where the infrastructure of the given network is built by FCM at the BS and the election process of CHs in each simulation round is conducted locally in each cluster instead of the BS. This is based on a new a multicriteria objective function where the network energy consumption, intracluster distance, and cluster-to-base station distance are main factors. Simulation results have shown that the proposed algorithm can improve network lifetime compared to popular existing algorithms such as C-FCM and LEACH. This improvement is based on different factors. Firstly, significant energy savings are achieved by the FCM-based clustering algorithm by discovering the most appropriate network infrastructure. Clusters are built with minimum distance from noncluster head nodes to their CHs and also minimum distance from CHs to their BS. Secondly, the decentralized technique proposed in our protocol leads to lower network overhead, which in turn lowers network energy expenditure. Thirdly, the adopted multicriteria objective function will always attempt to produce a set of good compromises or trade-offs, where the values of all the criteria are acceptable to the system requirements. Overall, the simulation results show that the proposed protocol achieves optimal network configuration, which reduces total network energy dissipation while at the same time increases network lifetime.

## Figures and Tables

**Table 1:** The settings for each simulation.

Parameters	Simulation	
	First simulation 100 m × 100 m	Second simulation 500 m × 500 m
Number of nodes	100	200
Base station location	(50, 175)	(500, 575)
Number of clusters	5	
Initial energy	2 J	
*E*_elec_	50 pJ/bit	
*E*_fs_	10 pJ/bit/m^2^	
*E*_mp_	0.0013 pJ/bit/m^4^	
*α*	0.75	
Data message *b*	500 bytes/message	
Control packet	25 bytes	
